# Effect of the gut microbiota–blood metabolite axis on anti-influenza IgG levels after vaccination: A Mendelian randomization study

**DOI:** 10.1097/MD.0000000000044100

**Published:** 2025-08-29

**Authors:** Dongmei Zhang, Fangyi He, Zhongpeng Zhao, Yuanqiang Zheng, Bochang Shi

**Affiliations:** a Xidian Group Hospital, Universal Hospitals Group, Xi’an, Shaanxi, China; b Tumen Community Health Service Center, Xi’an, Shaanxi, China; c School of Control Engineering, Northeastern University at Qinhuangdao, Qinhuangdao, Hebei, China; d Inner Mongolia Key Laboratory of Molecular Biology, Inner Mongolia Medical University, Hohhot, China.

**Keywords:** anti-influenza IgG level, gastrointestinal microbiome, Mendelian randomization analysis, metabolomics

## Abstract

While antibody responses to influenza viruses have been extensively studied, the immunogenicity of influenza vaccines remains highly variable among individuals. Growing evidence suggests that the gut microbiota (GM) and associated metabolites play a critical yet understudied role in shaping host immunity, including responses to vaccines. However, the mechanistic pathways linking microbial communities, blood metabolites, and influenza vaccine-induced antibody production remain poorly understood. This study investigates the GM–blood metabolite–antibody level axis to identify potential modulators of vaccine immunogenicity. In this study, 2-sample Mendelian randomization (MR) were conducted to identify causality. We performed 2-sample MR using genome-wide association study data from MiBioGen (GM, N = 14,306), Avon Longitudinal Study of Parents and Children (anti-influenza immunoglobulin G (IgG), N = 4735), and metabolite genome-wide association study (N = 7824). Instrumental variables (*P* < 5 × 10^-^⁵, *r*² < 0.01, *F* > 10) were analyzed via inverse-variance-weighted (IVW) regression with sensitivity analyses (MR-Egger, weighted median). Two-step MR assessed metabolite mediation effects. Our analysis revealed positive correlations between influenza virus subtype influenza A H1N1 virus IgG levels and the *Escherichia Shigella* genus, *Ruminococcaceae UCG002*, and *Ruminococcaceae UCG003* genera. Conversely, negative correlations were observed with the Erysipelotrichaceae family, Rhodospirillaceae family, *Barnesiella* genus, and *Eubacterium fissicatena* group. For influenza virus subtype influenza A H3N2 virus IgG antibodies, positive associations were identified with the Bacteroidales S24-7*group, Defluviitaleaceae family, *Adlercreutzia*, *Desulfovibrio*, *Eubacterium eligens* group, *Eubacterium rectale* group, and *Ruminococcaceae UCG014* genus, while negative associations were noted for the Negativicutes class and Selenomonadales order. Two-step MR analysis suggested mediation effects: the association between the Erysipelotrichaceae family and influenza A H1N1 virus IgG levels was partially mediated by bradykinin, des-Arg(9) (proportion mediated: 27.16%, *P* = .006). The link between *Ruminococcaceae UCG014* and influenza A H3N2 virus IgG levels was partially mediated by alanine (proportion mediated: 26.86%, *P* = .002). This study demonstrates the potential role of blood metabolites as mediators in modulating the impact of the GM on immunity to influenza.

## 1. Background

Influenza is a significant public health challenge, imposing a substantial burden of morbidity and mortality worldwide, resulting in approximately 3 to 5 million severe cases and 290,000 to 650,000 respiratory disease-related deaths annually. Vaccination remains the most effective and cost-efficient strategy for preventing influenza and controlling epidemics.^[1]^ When a host is infected with a virus or vaccinated with a virus-specific vaccine, B cells secrete immunoglobulin G (IgG) antibodies to provide specific immunity to suppress infection.^[2,3]^

Despite the WHO’s annual evaluation and updating of influenza vaccine strains, the vaccine’s efficacy is sometimes suboptimal. The seasonal influenza vaccine has generally ranged between 40% to 60% from 2004 to 2024, with the lowest efficacy recorded at only 10%.^[4]^ Similar trends are observed in recent studies from Europe and our country, where the vaccine efficacy ranges from 40% to 60%.^[5–9]^

Previous research has identified several key factors influencing the efficacy of the seasonal influenza vaccine, including the degree of match between the vaccine strain and the prevalent strain,^[10]^ pre-immunization exposure,^[11]^ age,^[12]^ immune status.^[13]^ Recent advances in research suggest that alterations in the gut microbiome (GM) and associated metabolic profiles significantly impact host immune status and specific IgG levels.^[14,15]^ However, the specific microorganisms and metabolites that support B cell responses remain incompletely identified. Therefore, further studies are needed to explore the effects of GM and their metabolites on IgG levels in response to influenza vaccines.

Mendelian randomization (MR) is a widely accepted method to control for potential confounders.^[16]^ With genetics measurement tools, this method can more reliably infer the causal relationship between the factor of interest and the disease, and avoid confounding factors as much as possible.^[17]^ Compared to traditional analytical methods, MR is well suited as a primary screening tool to infer causality between GM, learned metabolites, and the immune response to influenza.

This study aims to systematically evaluate the potential causal relationships between GM, their metabolites, and anti-influenza virus IgG levels using MR methods. Through this study, we aim to identify key microorganisms and metabolites that may enhance B cell-mediated immune responses, providing novel biomarkers and potential intervention targets to optimize influenza vaccine efficacy. This research will not only deepen our understanding of the role of GM in vaccine immunity but also lay the foundation for future microbiota-based immune enhancement strategies.

## 2. Methods

### 2.1. Study design

**Figure 1.**
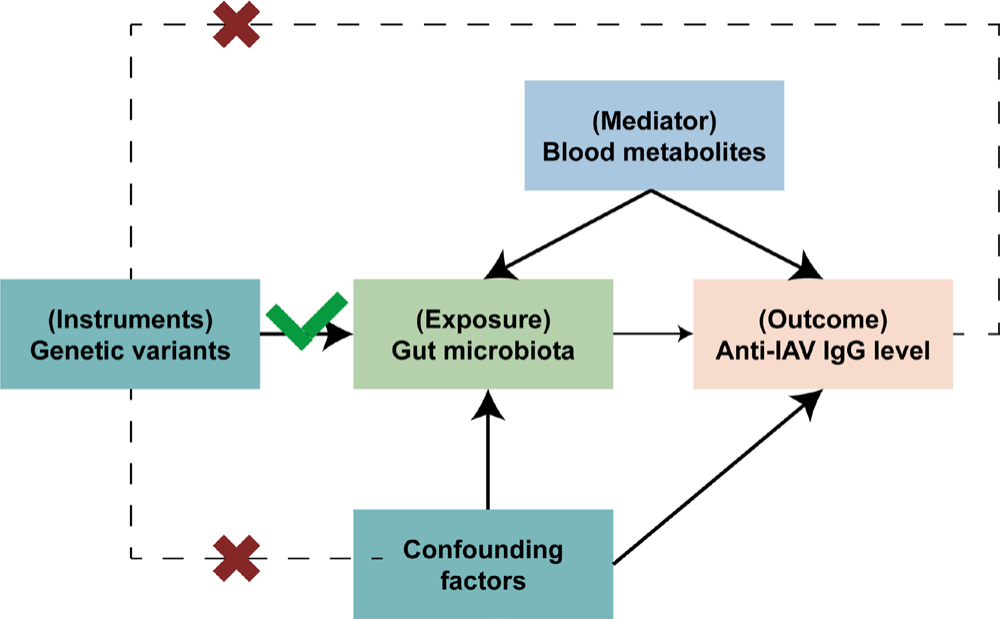
·Assumptions and design of the MR analyses and mediation MR analyses. Firstly, a 2-sample MR was performed to investigate the causal relationships between gut microbiota (exposure) and anti-influenza IgG level (outcome). Secondly, blood metabolites (mediator) were selected for subsequent mediation analyses. Finally, a 2-step MR analysis was conducted to detect potential mediating metabolites (step 1, the effect of gut microbiota on metabolites; step 2, the effect of metabolites on anti-influenza IgG level), followed by a validation analysis using 2-step MR. IgG = immunoglobulin G, MR = Mendelian randomization.

As shown in Figure 1, the study first used 2-sample MR to investigate the causal relationship between GM and anti-influenza IgG levels. Additionally, the causal effect of GM on blood metabolites was evaluated and the effect of these metabolites on anti-influenza IgG levels was then assessed. Finally, 2-step MR identify the mediation effect of blood metabolites in the relationship between GM and anti-influenza IgG levels.

### 2.2. Data sources

For anti-influenza virus IgG levels, summary genome-wide association study (GWAS) data from databases ieu-b-4903 and ieu-b-4904, sourced from the IEU Open GWAS Project, were analyzed, using a sample of 4735 individuals from the Avon Longitudinal Study of Parents and Children cohort.^[18]^ Plasma metabolites were examined in a cohort of 7824 Europeans, analyzing 452 plasma metabolites.^[19]^ Gut microbiota data were obtained from the MiBioGen consortium, which included genome-wide genotypes and 16S fecal microbiome data from 18,340 individuals across 24 cohorts, with 14,306 European individuals from 18 cohorts detailed in Table S1, Supplemental Digital Content, https://links.lww.com/MD/P792.

Our study used only published or publicly available GWAS summary data that has been approved by the applicable ethics and institutional review committee, and therefore does not require ethical approval.

### 2.3. Instrumental variables (IVs) screening and MR analysis

We extracted independent single nucleotide polymorphism (SNPs) that were strongly associated with exposure as IVs, filtered by a criterion of *P* < 5 × 10^–5^ in a 5000 kb window around leader SNPs that were less associated with other SNPs in the region (LD, *r*^2^ < 0. 01). Last but not least, in order to determine the statistical of the selected SNPs, the *F*-statistic was calculated based on the formula *F* = *R*^2^(N - *k* - 1)/[(1 – *R*^2^)*k*], where *R*^2^ is the proportion of variability explained by each SNP, N is the GWAs sample size, and *k* is the number of SNPs. A *F*-statistic is < 10 indicates that the IV is a weak instrument and is therefore not used. This rigorous screening ensured the use of strong, reliable IVs, enhancing the study’s findings on the causal relationship between GM, blood metabolites, and anti-influenza IgG levels.

Palindromic SNPs were excluded to avoid strand alignment ambiguity. To explore the causal effects of GM features, IgG levels, and metabolites, we conducted 2-sample MR analyses in the TwoSampleMR R package (University of Bristol, MRC Integrative Epidemiology Unit [Opensource academic project], Bristol, United Kingdom): fixed-effects inverse-variance-weighted (IVW (fe)), multiplicative random-effects IVW, weighted median, and MR-Egger regression. We prioritized IVW (fe) results in the absence of heterogeneity or pleiotropy, MR-Egger regression to assess directional pleiotropy, and weighted median to address heterogeneity. Causal effects were estimated using IVW (fe) and MR-Egger regression (*P* < .05), with FDR correction to control false positives. Heterogeneity and pleiotropy were assessed using MR-Egger intercept and Cochran *Q* test, including only exposures with no pleiotropy (*P*-value > .05) and no significant heterogeneity (*Q*-value > 0.05).

### 2.4. Mediation analyses link “GM–metabolites–IgG level”

Univariable MR initially estimated each exposure’s total effect, while 2-step MR dissected these effects into direct and mediated components. Initially, we estimated the total effect (β1) using univariable MR, which assesses the overall impact of exposures like GM on IgG levels. Direct effects were evaluated through the controlled direct effect (β2), isolating the influence of metabolites on IgG levels adjusted for GM. Indirect effects, measured by the product of coefficients method (α × β2*), revealed how GM affects IgG levels via blood metabolites, and vice versa. The proportion mediated, calculated as ([α × β2*]/β1), provided insights into the extent to which metabolites mediated the relationship between GM and IgG levels, and vice versa.

### 2.5. Software and pre-registration

R (version 4.1.2; The R Foundation for Statistical Computing, Vienna, Austria) serves as the primary environment for statistical computations and data manipulation. Key R packages include TwoSampleMR for 2-sample MR analyses, tidyverse for data manipulation and visualization, ggplot2 for creating graphs, purrr for functional programming, and data.table for efficient data handling.

### 2.6. Ethics

Individual studies within each GWAS received approval from the relevant Institutional Review Board, and informed consent was obtained from the participants or a caregiver, legal guardian, or other proxy.

## 3. Results

### 3.1. GM and anti-influenza virus IgG level

**Figure 2.**
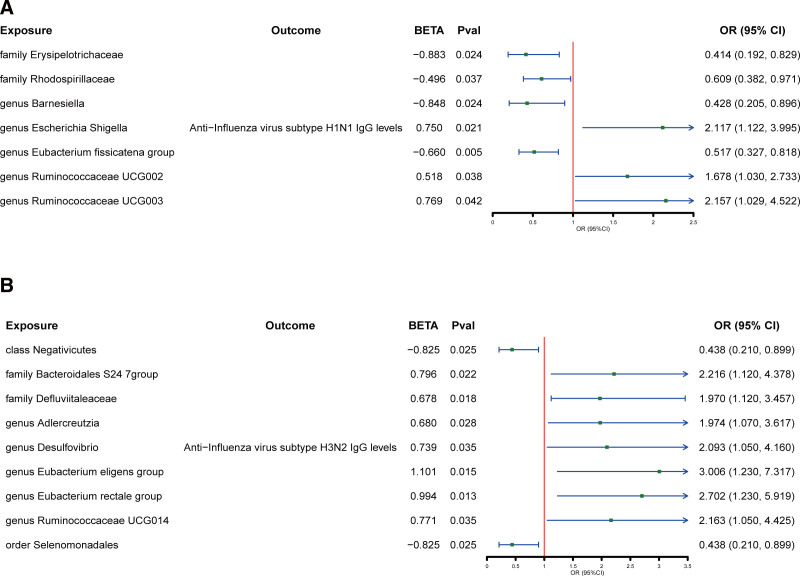
The causal effect of gut microbiota on anti-influenza IgG level. (A) Exposure factors with causal effects on anti-H1N1 IgG (gut microbiota). (B) Exposure factors with causal effects on anti-H3N2 IgG (gut microbiota). The green point means the effect (OR). CI = confidence interval, IgG = immunoglobulin G, OR = odds ratio.

When evaluating the causal effects of GM on anti-influenza virus IgG levels, 2 families and 2 genera were negatively and 3 genera were positively associated with anti-influenza A H1N1 virus (H1N1) IgG levels using 3 methods (Fig. 2A and Table S2, Supplemental Digital Content, https://links.lww.com/MD/P792). Additionally, 1 class was negatively associated with anti-H1N1 IgG levels, whereas 2 families and 5 genera were positively associated with anti-influenza A H3N2 virus (H3N2) IgG levels (Fig. 2B and Table S3, Supplemental Digital Content, https://links.lww.com/MD/P792). Among these significant taxa, the family Erysipelotrichaceae, genus *Eubacterium fissicatena* group, genus *Ruminococcaceae*, class Negativicutes, family Defluviitaleaceae, genus *Eubacterium eligens* group, and genus *Eubacterium rectale* group are part of the *Bacillota phylum*; the family Rhodospirillaceae, genus *Escherichia Shigella*, and genus *Desulfovibrio* are part of the *Pseudomonadota phylum*; the genus *Barnesiella* and order Bacteroidales S24-7 are part of the Bacteroidota phylum; and the genus *Adlercreutzia* are part of the *Actinomycetota phylum*.

Notably, the genus *Ruminococcaceae UCG003* exhibited a particularly strong positive association with anti-H1N1 IgG levels (odds ratio [OR] = 2·157, *P* = 0·042), suggesting a protective role in patients. Conversely, the family *Erysipelotrichaceae* showed negative associations with anti-H1N1 IgG levels (OR = 0·414, *P* = 0·024), indicating a potential role in reduced IgG levels. Strong positive correlation between the genus *Eubacterium* and anti-H1N1 IgG levels (OR = 3·006, *P* = 0·015), suggesting a protective effect. Additionally, the class Negativicutes showed negative associations with anti-H3N2 IgG levels (OR = 0·438, *P* = 0·025), indicating a potential role in low IgG levels.

Heterogeneity analyses using Cochran *Q* test indicated no significant heterogeneity among IVs used in MR analyses, suggesting consistent effects across different genetic variants. Furthermore, MR-Egger intercept tests showed non-significant results, indicating the absence of horizontal pleiotropy and supporting that genetic variants do not affect outcomes through pathways unrelated to the exposure of interest. Table S4, Supplemental Digital Content, https://links.lww.com/MD/P792 provided additional detailed results from sensitivity and pleiotropy tests, confirming the study’s robustness and reliability in establishing causal relationships between specific GM families and genera and anti-H1N1 IgG levels.

### 3.2. Blood metabolites and anti-influenza virus IgG level

Glycerate, heptanoate, betaine, DSGEGDFXAEGGGVR^*^, ADSGEGDFXAEGGGVR^*^, 1-arachidonoylglycerophosphoinositol^*^, des-Arg9-bradykinin (DABK), 2-palmitoylglycerophosphocholine^*^, and the free cholesterol to total lipids ratio in large low-density lipoprotein (LDL) had a negative correlation with anti-H1N1 IgG levels. Conversely, alanine, apolipoprotein A-I, free cholesterol, total cholesterol in HDL, concentration of very low-density lipoprotein particles, and triglycerides (TG) in very large HDL had a positive correlation with anti-H1N1 IgG levels (Table S5, Supplemental Digital Content, https://links.lww.com/MD/P792).

The results also showed that guanosine, myo-inositol, indoleacetate, alanine, phenylalanylleucine, acetoacetate, the ratio of bisallylic groups to total fatty acids, citrate, lactate, acetone, and cholesterol in medium HDL were negatively correlated with anti-H3N2 IgG levels. However, indolepropionate, MDC: %CD1c+, and TG in medium LDL were positively correlated with anti-H3N2 IgG levels (Table S6, Supplemental Digital Content, https://links.lww.com/MD/P792).

All *P*-values for Cochran *Q* test and MR-Egger intercept test were >.05, indicating the absence of heterogeneity and horizontal multiplicity.

### 3.3. Mediation analyses

**Figure 3.**
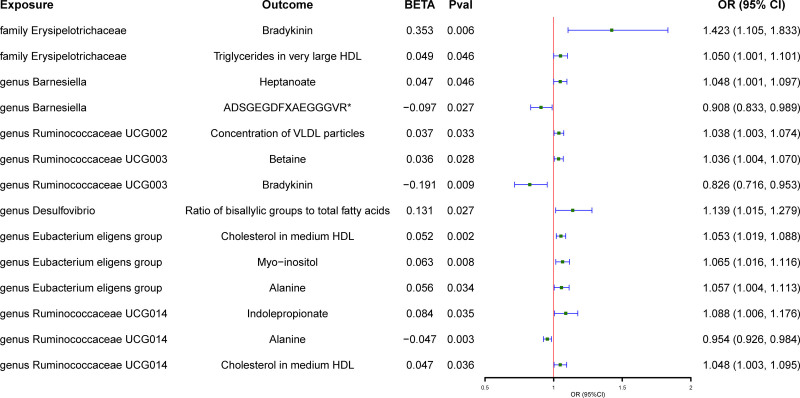
The causal effect of blood metabolites on gut microbiota. The green point means the effect (OR). OR = odds ratio.

**Figure 4.**
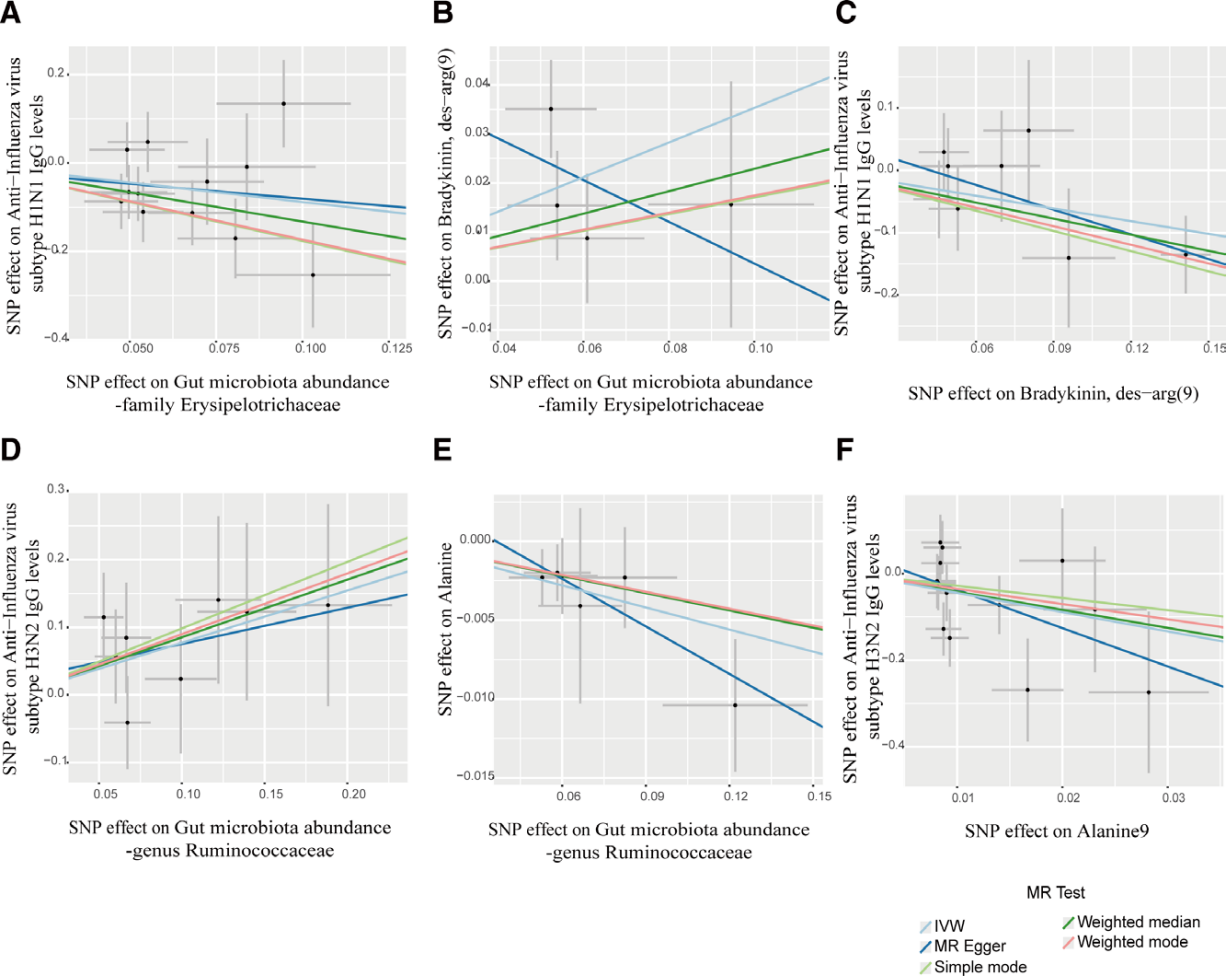
(A–F) Scatter plots of the above results indicate stability.

**Figure 5.**
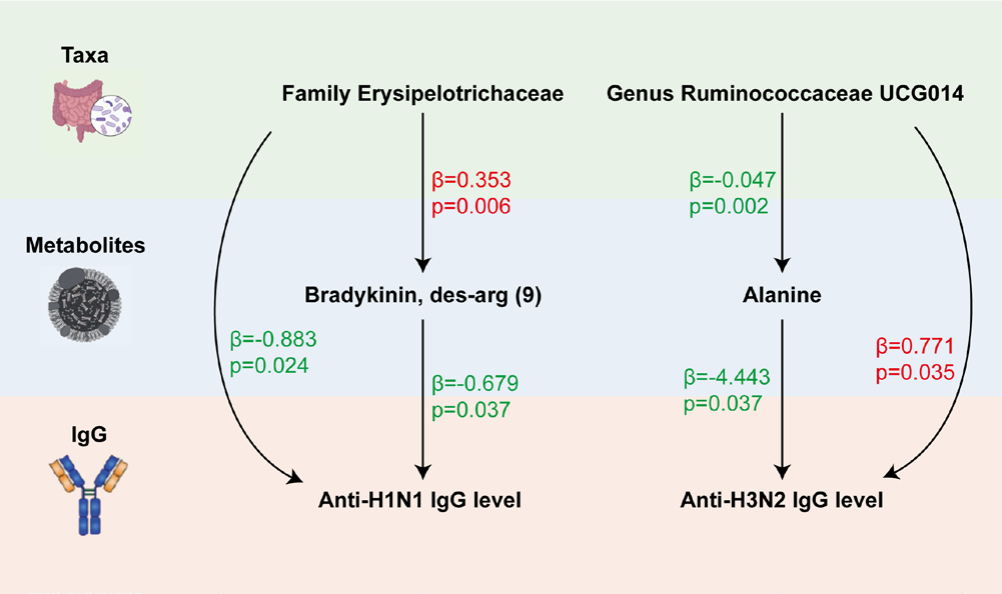
MR analyses show causal effects of blood metabolites on gut microbiota and anti-influenza IgG level. The diagram displays the mediation mode of “gut microbiota–blood metabolites–anti-influenza IgG level” in 2-step Mendelian randomization. Beta values (β) indicate the causal effect estimates using the inverse-variance-weighted method (truncated at *P* < .05). Characters colored in red and green signify positive and negative associations, respectively.

In the 2-step MR, 15 blood metabolites were causally associated with anti-H1N1 IgG levels, and 14 blood metabolites were causally associated with anti-H3N2 IgG levels (Fig. 3). Among the 18 taxa causally associated with IgG levels, 2 were significantly associated with the above 2 metabolites (Fig. 4 and Table S7, Supplemental Digital Content, https://links.lww.com/MD/P792). The family Erysipelotrichaceae had a detrimental effect on anti-H1N1 IgG levels (OR 1.423, 95% CI: 1.105–1.833, *P* = .006) by increasing DABK levels. Conversely, the genus *Ruminococcaceae UCG014* promoted anti-H3N2 IgG levels (OR 0.954, 95% CI: 0.926–0.984, *P* = .002) by downregulating alanine levels (Fig. 5). The *Q*-statistics indicated no notable heterogeneity (*P* > .05).

The mediation analysis conducted in the study has unveiled significant roles for specific metabolites in mediating the effects of GM on anti-H1N1 and anti-H3N2 IgG levels (Table 1). For anti-H1N1 IgG levels, it was found that 27.16% of the effect of the family Erysipelotrichaceae through the mediation of DABK levels. This highlights a pathway through which Erysipelotrichaceae influences anti-H1N1 IgG levels via its impact on DABK. Similarly, for anti-H3N2 IgG levels, genus *Ruminococcaceae UCG014* was shown to affect IgG levels, with 26·86% of this effect mediated through alanine levels.

**Table 1:** Two-step Mendelian randomization.

Exposure	Mediation	Total effect (β1*)	α	β2*	Indirect effect (α×β2*)	Mediation effect/total effect
f_Erysipelotrichaceae	Bradykinin, des-Arg(9)	-0.88294	0.353039	-0.67931	-0.23982	0.271619
g_Ruminococcaceae UCG014	Alanine	0.771466	-0.04664	-4.44278	0.207226	0.268613

β1* and β2* represent the controlled direct effects of each pair of bacteria and metabolite on outcomes after adjusting for each other. α is the causal effect of exposure on mediator; indirect effect (α×β2*) is the effect of exposure on IgG level via corresponding mediator; β1 is the total effect of exposure on stroke; proportion mediated is calculated as the “indirect effect/total effect.” The prefix “o_/f_/g_” represents order/family/genus respectively.

## 4. Discussion

Influenza viruses, including seasonal strains like H1N1, H3N2, and influenza B, cause significant respiratory infections globally, leading to 3 to 5 million severe cases and 290,000 to 650,000 deaths annually, as reported by the World Health Organization.^[20]^ Avian influenza viruses, such as H5N1 and H7N9, also contribute to zoonotic infections.^[21,22]^ Occasionally, animal influenza viruses cross species barriers through reassortment events, causing antigenic shifts that can lead to pandemics.^[23,24]^ These pandemics, such as the 1918 H1N1 outbreak which resulted in an estimated 40 million deaths,^[25]^ often exhibit higher morbidity and mortality rates than seasonal epidemics due to a lack of preexisting immunity.

Neutralizing antibodies from influenza vaccination are critical in combating outbreaks, with those targeting the viral surface glycoprotein hemagglutinin being particularly protective against infection.^[26]^ Recent advancements in single-cell antibody analysis have significantly enhanced our understanding of the antibody response to influenza.^[27]^ However, substantial knowledge gaps remain regarding how to influence antibody dynamics rationally during vaccination. Understanding the mechanisms behind long-lived immunity is essential for designing vaccines that provide enduring protection.

The study evaluated the causal relationship between GM taxa and anti-influenza IgG levels, identifying 9 GM taxa associated with H1N1 IgG levels and 7 GM taxa associated with H3N2 IgG levels. It revealed potential mechanisms through 2 blood metabolites linked to GM taxa and anti-influenza IgG levels. Specifically, the family Erysipelotrichaceae was found to negatively affect anti-H1N1 IgG levels by increasing DABK, while the genus *Ruminococcaceae UCG-014* positively affected anti-H3N2 IgG levels by decreasing alanine. These findings suggest possible pathways through which GM may influence immune responses to influenza. Interestingly, the overlap between Tu et al findings^[28]^ and our study further support the potential role of specific GM taxa in modulating vaccine-induced immunity. For instance, both studies highlight the importance of Ruminococcaceae-related taxa in enhancing anti-influenza IgG levels. Additionally, the involvement of Erysipelotrichaceae in our study aligns with Tu et al emphasis on the impact of specific microbial families on immune regulation. These shared findings suggest that Ruminococcaceae and Erysipelotrichaceae may serve as key microbial targets for optimizing influenza vaccine efficacy.

Previous studies have shown that the composition and diversity of the host microbiota undergoes significant changes, which can directly or indirectly influence immune system regulation.^[29,30]^ Erysipelotrichaceae have been observed in the cecum and feces, with certain genera linked to inflammatory gastrointestinal disorders or diarrhea.^[31]^ Brittany study further elucidated the relationship between Erysipelotrichaceae and exposure to SARS-CoV-2 and influenza A virus using aged glutathione modeling.^[32]^ In the small intestine, unclassified Erysipelotrichaceae were enriched in both SARS-CoV-2 and FLUAV groups compared to mock groups, with notable peaks at 3 days post-challenge. Similarly, in feces, unclassified Erysipelotrichaceae were enriched in both SARS-CoV-2 and FLUAV groups compared to mock and pre-challenge groups. Additionally, in abiotic models, Erysipelotrichaceae taxa have been associated with severe malnutrition and IgA responses.^[33]^

The study is the first to report that the family Erysipelotrichaceae regulates DABK, part of the kallikrein–kinin system,^[34,35]^ where kallikrein converts kininogen into bradykinin (BK), inducing vasodilation and anti-ischemic effects via the kinin B2 receptor, and carboxypeptidase N metabolizes BK into DABK,^[35,36]^ causing vasoconstriction and cell proliferation via the kinin B1 receptor.^[36,37]^ Fernandes et al clinical study showed that BK levels inversely correlate with homeostasis model assessment, systolic blood pressure, and C-reactive protein, while DABK levels positively correlate with these parameters.^[38]^ Additionally, Xu et al reported that DABK levels increased during early anti-infective therapy and decreased during consolidation and post-therapy, indicating a shift from active disease to chronic inflammation to a disease-free state.^[39,40]^ All of the above evidence suggests that DABK is a detrimental factor that may directly or indirectly suppress humoral immunity and antibody production through pro-inflammatory and other effects.

Another crucial pathway involves the downregulation of alanine by *Ruminococcaceae UCG-014*. *Ruminococcaceae UCG-014* are recognized as beneficial short-chain fatty acid-producing bacteria.^[41]^ The abundances of *Ruminococcaceae UCG-014* are negatively correlated with serum TG, total cholesterol, and LDL-C. Furthermore, *Ruminococcaceae UCG-014* has shown significant associations with interleukin-6, serotonin, and microbial metabolism pathways.^[42]^

We found that high alanine levels appear to be associated with lower H3N2 antibody levels. Although direct evidence linking alanine to influenza antibody levels is lacking, previous studies suggest that abnormalities in alanine metabolism may lead to its accumulation in the blood. During lung injury, epithelial and other cells may experience energetic stress,^[43,44]^ necessitating rapid and effective energy production pathways for survival.^[45]^ Glucose and alanine serve as precursors for pyruvate metabolism, while amino acid and fatty acid catabolism generate substrates for the tricarboxylic acid cycle and energy production. Decreased concentrations of these metabolites in the serum of patients may indicate increased utilization of aerobic metabolism via the Krebs cycle.^[46]^ These findings align with recent reports by Banoei,^[47]^ which highlighted that disruptions in amino acid metabolism and gluconeogenesis pathways may predict poor prognosis in H1N1 infection. Similar results have been observed in animal models of acute lung injury. Chen^[48]^ reported energy disorders in mouse serum during H1N1-induced pneumonia, which may partially explain the impact of high alanine levels on antibody production. These findings underscore the intricate link between metabolic regulation and immune response, suggesting that alanine metabolic abnormalities could impair effective immune defense mechanisms against influenza.

No previous study, to our knowledge, has directly linked Erysipelotrichaceae and Ruminococcaceae UCG-014 to the kallikrein–kinin system and alanine metabolism. Our mediation analyses provide genetic evidence for underlying relationships between genetic modifications, blood metabolites, and influenza IgG levels. Such findings emphasize the promise of purposefully modulating GM as a strategy to improve influenza infection outcomes and antibody levels, and warrant further investigation of the interactions between GM, the immune response to infection, and metabolite regulation in future studies.

## 5. Conclusion

This study provides the first comprehensive assessment of the causal relationship between GM, blood metabolites, and anti-influenza IgG levels. Our findings emphasize the significance of elucidating the intrinsic link between genetic modification and IgG levels, providing new perspectives for both microbiome-based influenza therapies and metabolite-targeted interventions.

## Author contributions

**Conceptualization:** Dongmei Zhang, Bochang Shi.

**Data curation:** Fangyi He, Bochang Shi.

**Formal analysis:** Zhongpeng Zhao.

**Funding acquisition:** Zhongpeng Zhao.

**Investigation:** Yuanqiang Zheng.

**Methodology:** Fangyi He, Bochang Shi.

**Resources:** Dongmei Zhang.

**Supervision:** Dongmei Zhang.

**Software:** Bochang Shi.

**Validation:** Zhongpeng Zhao.

**Visualization:** Bochang Shi.

**Writing – original draft:** Fangyi He, Bochang Shi.

**Writing – review & editing:** Fangyi He, Bochang Shi.

## Supplementary Material


