# Supplementary material for: Effect of the gut microbiota–blood metabolite axis on anti-influenza IgG levels after vaccination: A Mendelian randomization study
Source: Medicine (Baltimore). 2025 Aug 29;104(35):e44100. doi: 10.1097/MD.0000000000044100 (PMC12401250; doi:10.1097/MD.0000000000044100)
Supplement: Supplementary file 1 [file medi-104-e44100-s001.docx]

| **Supplementary Table 1. Characteristics of summary genome-wide association studies.** | | | | |  |
| --- | --- | --- | --- | --- | --- |
|  | **Trait** | **Sample size** | **Ancestry** | **Year** | **Study reference or description URL** |
| **Exposure** | Gut microbiota | 18340 | Mixed (78% European) | 2021 | Nat Genet. 2021; 53:156-165. https://doi.org/10.1038/s41588-020-00763-1 |
| **Outcome** | Anti-influenza IgG level | 14901 | European | 2021 | Front Immunol. 2021 Nov 4;12:727457. doi: 10.3389/fimmu.2021.727457. PMID: 34804013; PMCID: PMC8599591. |
| **Mediator** | Blood metabolites | 7824 | European | 2014 | Nat Genet. 2014 Jun;46(6):543-550. doi: 10.1038/ng.2982. Epub 2014 May 11. PMID: 24816252; PMCID: PMC4064254. |

| **Supplementary Table 2. Mendelian randomization models estimate the causal effects of gut microbiota on anti-influenza H1N1 IgG level** | | | | | | | | | |  |  |
| --- | --- | --- | --- | --- | --- | --- | --- | --- | --- | --- | --- |
|  |  |  |  |  |  |  |  |  |  |  |  |
| Exposure | Outcome | Method | Nsnp | BETA | SE | Pval | OR (95% CI) | Pleio_egger_intercept | Pleio_Pval | Het_Q | Het_Q_pval |
| family Erysipelotrichaceae | Anti-Influenza virus subtype H1N1 IgG levels | MR Egger | 12 | -0.674168 | 1.665727 | 0.694199 | 0.510 (0.019, 13.339) | -0.01338 | 0.899679 | 13.85709 | 0.17961 |
|  |  | Weighted median | 12 | -1.325154 | 0.498172 | 0.007813 | 0.266 (0.103, 0.688) | |  | 13.88026 | 0.23969 |
|  |  | Inverse variance weighted | 12 | -0.882937 | 0.39107 | 0.023961 | 0.414 (0.192, 0.890) | |  |  |  |
| family Rhodospirillaceae |  | MR Egger | 15 | -1.468082 | 1.215125 | 0.2485 | 0.230 (0.021, 2.493) | 0.094733 | 0.429086 | 15.19442 | 0.295373 |
|  |  | Weighted median | 15 | -0.26936 | 0.309514 | 0.384154 | 0.764 (0.416, 1.401) | |  | 15.97306 | 0.315022 |
|  |  | Inverse variance weighted | 15 | -0.496016 | 0.238239 | 0.037341 | 0.609 (0.382, 0.971) | |  |  |  |
| genus Barnesiella |  | MR Egger | 12 | -1.970499 | 1.172705 | 0.123818 | 0.139 (0.014, 1.388) | 0.094249 | 0.336009 | 14.92487 | 0.134825 |
|  |  | Weighted median | 12 | -0.969335 | 0.442094 | 0.028336 | 0.379 (0.159, 0.902) | |  | 16.44934 | 0.125252 |
|  |  | Inverse variance weighted | 12 | -0.84799 | 0.376727 | 0.024389 | 0.428 (0.205, 0.896) | |  |  |  |
| genus Escherichia Shigella |  | MR Egger | 9 | -0.499871 | 0.907643 | 0.598943 | 0.607 (0.102, 3.593) | 0.101996 | 0.183921 | 1.044539 | 0.994078 |
|  |  | Weighted median | 9 | 0.634884 | 0.420565 | 0.131146 | 1.887 (0.827, 4.302) | |  | 3.217839 | 0.919952 |
|  |  | Inverse variance weighted | 9 | 0.749983 | 0.324084 | 0.020659 | 2.117 (1.122, 3.996) | |  |  |  |
| genus Eubacterium fissicatena group |  | MR Egger | 6 | -2.594328 | 1.230632 | 0.102707 | 0.075 (0.007, 0.833) | 0.254206 | 0.184558 | 1.341943 | 0.85422 |
|  |  | Weighted median | 6 | -0.669741 | 0.307267 | 0.029282 | 0.512 (0.280, 0.935) | |  | 3.90623 | 0.562994 |
|  |  | Inverse variance weighted | 6 | -0.659587 | 0.233889 | 0.004801 | 0.517 (0.327, 0.818) | |  |  |  |
| genus Ruminococcaceae UCG002 |  | MR Egger | 20 | 1.278964 | 0.663483 | 0.069829 | 3.593 (0.979, 13.189) | -0.061698 | 0.231662 | 13.74011 | 0.745869 |
|  |  | Weighted median | 20 | 0.351302 | 0.354093 | 0.321139 | 1.421 (0.710, 2.844) | |  | 15.27247 | 0.70513 |
|  |  | Inverse variance weighted | 20 | 0.51764 | 0.248918 | 0.037566 | 1.678 (1.030, 2.733) | |  |  |  |
| genus Ruminococcaceae UCG003 |  | MR Egger | 8 | -0.179888 | 1.149972 | 0.880826 | 0.835 (0.088, 7.957) | 0.07236 | 0.416146 | 1.278673 | 0.972821 |
|  |  | Weighted median | 8 | 0.666728 | 0.476398 | 0.161658 | 1.948 (0.766, 4.955) | |  | 2.041091 | 0.957531 |
|  |  | Inverse variance weighted | 8 | 0.76851 | 0.377742 | 0.041903 | 2.157 (1.029, 4.522) | |  |  |  |

| **Supplementary Table 3. Mendelian randomization models estimate the causal effects of gut microbiota on anti-influenza H3N2 IgG level** | | | | | | | | | | | |
| --- | --- | --- | --- | --- | --- | --- | --- | --- | --- | --- | --- |
| Exposure | Outcome | Method | Nsnp | BETA | SE | Pval | OR (95% CI) | Pleio_egger_intercept | Pleio_Pval | Het_Q | Het_Q_pval |
| class Negativicutes | Anti-Influenza virus subtype H3N2 IgG levels | MR Egger | 11 | 0.695618 | 1.122477 | 0.550809 | 2.005 (0.22, 18.096) | -0.10423 | 0.185647 | 3.561437 | 0.937841 |
|  |  | Weighted median | 11 | -1.05865 | 0.506554 | 0.036626 | 0.347 (0.12, 0.936) | |  | 5.615121 | 0.846497 |
|  |  | Inverse variance weighted | 11 | -0.82472 | 0.366682 | 0.024504 | 0.438 (0.21, 0.899) | |  |  |  |
| family Bacteroidales S24 7group |  | MR Egger | 6 | 1.024192 | 1.355761 | 0.492031 | 2.785 (0.19, 39.705) | -0.02481 | 0.870098 | 0.951611 | 0.917042 |
|  |  | Weighted median | 6 | 0.958268 | 0.419859 | 0.022468 | 2.607 (1.14, 5.937) | |  | 0.981991 | 0.964005 |
|  |  | Inverse variance weighted | 6 | 0.795776 | 0.347409 | 0.021987 | 2.216 (1.12, 4.378) | |  |  |  |
| family Defluviitaleaceae |  | MR Egger | 8 | 1.556945 | 0.984563 | 0.164881 | 4.744 (0.68, 32.678) | -0.10755 | 0.386669 | 5.474227 | 0.484577 |
|  |  | Weighted median | 8 | 0.230185 | 0.400593 | 0.565554 | 1.259 (0.57, 2.760) | |  | 6.345332 | 0.500054 |
|  |  | Inverse variance weighted | 8 | 0.677909 | 0.286922 | 0.018143 | 1.970 (1.12, 3.457) | |  |  |  |
| genus Adlercreutzia |  | MR Egger | 8 | 0.795467 | 1.376413 | 0.584335 | 2.215 (0.14, 32.892) | -0.01049 | 0.934297 | 3.713354 | 0.715401 |
|  |  | Weighted median | 8 | 0.485686 | 0.395352 | 0.219263 | 1.625 (0.74, 3.527) | |  | 3.720742 | 0.811322 |
|  |  | Inverse variance weighted | 8 | 0.680173 | 0.308954 | 0.027698 | 1.974 (1.07, 3.617) | |  |  |  |
| genus Desulfovibrio |  | MR Egger | 7 | 0.525712 | 1.30003 | 0.702647 | 1.692 (0.13, 21.623) | 0.021814 | 0.871528 | 1.983239 | 0.851459 |
|  |  | Weighted median | 7 | 0.703392 | 0.462488 | 0.128288 | 2.021 (0.81, 5.002) | |  | 2.012206 | 0.918573 |
|  |  | Inverse variance weighted | 7 | 0.738786 | 0.350377 | 0.034984 | 2.093 (1.05, 4.160) | |  |  |  |
| genus Eubacterium eligens group |  | MR Egger | 7 | -2.08813 | 1.708657 | 0.276139 | 0.124 (0.00, 3.528) | 0.263108 | 0.111572 | 2.852536 | 0.722708 |
|  |  | Weighted median | 7 | 0.828076 | 0.607614 | 0.172935 | 2.289 (0.69, 7.531) | |  | 6.575241 | 0.361919 |
|  |  | Inverse variance weighted | 7 | 1.100734 | 0.453818 | 0.015288 | 3.006 (1.23, 7.317) | |  |  |  |
| genus Eubacterium rectale group |  | MR Egger | 9 | 1.486458 | 1.375128 | 0.315543 | 4.421 (0.29, 65.478) | -0.03249 | 0.719161 | 6.93007 | 0.436197 |
|  |  | Weighted median | 9 | 0.986384 | 0.575503 | 0.086537 | 2.682 (0.86, 8.284) | |  | 7.070277 | 0.52907 |
|  |  | Inverse variance weighted | 9 | 0.993838 | 0.400183 | 0.013011 | 2.702 (1.23, 5.919) | |  |  |  |
| genus Ruminococcaceae UCG014 |  | MR Egger | 8 | 0.535541 | 0.918327 | 0.58102 | 1.708 (0.28, 10.334) | 0.021764 | 0.788877 | 3.583811 | 0.732788 |
|  |  | Weighted median | 8 | 0.851888 | 0.475979 | 0.073493 | 2.344 (0.92, 5.958) | |  | 3.662213 | 0.817757 |
|  |  | Inverse variance weighted | 8 | 0.771466 | 0.365222 | 0.034659 | 2.163 (1.05, 4.425) | |  |  |  |
| order Selenomonadales |  | MR Egger | 11 | 0.695618 | 1.122477 | 0.550809 | 2.005 (0.22, 18.096) | -0.10423 | 0.185647 | 3.561437 | 0.937841 |
|  |  | Weighted median | 11 | -1.05865 | 0.484778 | 0.028978 | 0.347 (0.13, 0.897) | |  | 5.615121 | 0.846497 |
|  |  | Inverse variance weighted | 11 | -0.82472 | 0.366682 | 0.024504 | 0.438 (0.21, 0.899) | |  |  |  |

| **Supplementary Table 4. Pleiotropy of all Mendelian randomization results** | | | |  |  |  |
| --- | --- | --- | --- | --- | --- | --- |
| Exposure | Outcome | Method | Pleio_egger_intercept | Pleio_Pval | Het_Q | Het_Q_pval |
| family Erysipelotrichaceae | Anti-Influenza virus subtype H1N1 IgG levels | MR Egger | -0.01338 | 0.899679 | 13.85709 | 0.17961 |
|  |  | Weighted median | |  | 13.88026 | 0.23969 |
|  |  | Inverse variance weighted | |  |  |  |
| family Rhodospirillaceae |  | MR Egger | 0.094733 | 0.429086 | 15.19442 | 0.295373 |
|  |  | Weighted median | |  | 15.97306 | 0.315022 |
|  |  | Inverse variance weighted | |  |  |  |
| genus Barnesiella |  | MR Egger | 0.094249 | 0.336009 | 14.92487 | 0.134825 |
|  |  | Weighted median | |  | 16.44934 | 0.125252 |
|  |  | Inverse variance weighted | |  |  |  |
| genus Escherichia Shigella |  | MR Egger | 0.101996 | 0.183921 | 1.044539 | 0.994078 |
|  |  | Weighted median | |  | 3.217839 | 0.919952 |
|  |  | Inverse variance weighted | |  |  |  |
| genus Eubacterium fissicatena group |  | MR Egger | 0.254206 | 0.184558 | 1.341943 | 0.85422 |
|  |  | Weighted median | |  | 3.90623 | 0.562994 |
|  |  | Inverse variance weighted | |  |  |  |
| genus Ruminococcaceae UCG002 |  | MR Egger | -0.0617 | 0.231662 | 13.74011 | 0.745869 |
|  |  | Weighted median | |  | 15.27247 | 0.70513 |
|  |  | Inverse variance weighted | |  |  |  |
| genus Ruminococcaceae UCG003 |  | MR Egger | 0.07236 | 0.416146 | 1.278673 | 0.972821 |
|  |  | Weighted median | |  | 2.041091 | 0.957531 |
|  |  | Inverse variance weighted | |  |  |  |
| class Negativicutes | Anti-Influenza virus subtype H3N2 IgG levels | MR Egger | -0.10423 | 0.185647 | 3.561437 | 0.937841 |
|  |  | Weighted median | |  | 5.615121 | 0.846497 |
|  |  | Inverse variance weighted | |  |  |  |
| family Bacteroidales S24 7group |  | MR Egger | -0.02481 | 0.870098 | 0.951611 | 0.917042 |
|  |  | Weighted median | |  | 0.981991 | 0.964005 |
|  |  | Inverse variance weighted | |  |  |  |
| family Defluviitaleaceae |  | MR Egger | -0.10755 | 0.386669 | 5.474227 | 0.484577 |
|  |  | Weighted median | |  | 6.345332 | 0.500054 |
|  |  | Inverse variance weighted | |  |  |  |
| genus Adlercreutzia |  | MR Egger | -0.01049 | 0.934297 | 3.713354 | 0.715401 |
|  |  | Weighted median | |  | 3.720742 | 0.811322 |
|  |  | Inverse variance weighted | |  |  |  |
| genus Desulfovibrio |  | MR Egger | 0.021814 | 0.871528 | 1.983239 | 0.851459 |
|  |  | Weighted median | |  | 2.012206 | 0.918573 |
|  |  | Inverse variance weighted | |  |  |  |
| genus Eubacterium eligens group |  | MR Egger | 0.263108 | 0.111572 | 2.852536 | 0.722708 |
|  |  | Weighted median | |  | 6.575241 | 0.361919 |
|  |  | Inverse variance weighted | |  |  |  |
| genus Eubacterium rectale group |  | MR Egger | -0.03249 | 0.719161 | 6.93007 | 0.436197 |
|  |  | Weighted median | |  | 7.070277 | 0.52907 |
|  |  | Inverse variance weighted | |  |  |  |
| genus Ruminococcaceae UCG014 |  | MR Egger | 0.021764 | 0.788877 | 3.583811 | 0.732788 |
|  |  | Weighted median | |  | 3.662213 | 0.817757 |
|  |  | Inverse variance weighted | |  |  |  |
| order Selenomonadales |  | MR Egger | -0.10423 | 0.185647 | 3.561437 | 0.937841 |
|  |  | Weighted median | |  | 5.615121 | 0.846497 |
|  |  | Inverse variance weighted | |  |  |  |
| family Erysipelotrichaceae | Bradykinin | MR Egger | 0.046139 | 0.383765 | 2.667637 | 0.263469 |
|  |  | Weighted median | |  | 4.300879 | 0.230754 |
|  |  | Inverse variance weighted | |  |  |  |
| family Erysipelotrichaceae | Triglycerides in very large HDL | MR Egger | 0.00787 | 0.235246 | 11.98084 | 0.365085 |
|  |  | Weighted median | |  | 13.69819 | 0.320395 |
|  |  | Inverse variance weighted | |  |  |  |
| genus Barnesiella | Heptanoate | MR Egger | 0.004216 | 0.550369 | 1.625062 | 0.443734 |
|  |  | Weighted median | |  | 2.131855 | 0.545495 |
|  |  | Inverse variance weighted | |  |  |  |
| genus Barnesiella | ADSGEGDFXAEGGGVR* | MR Egger | 0.009154 | 0.389262 | 0.188702 | 0.909963 |
|  |  | Weighted median | |  | 1.378498 | 0.710583 |
|  |  | Inverse variance weighted | |  |  |  |
| genus Ruminococcaceae UCG002 | Concentration of VLDL particles | MR Egger | -0.00109 | 0.764665 | 25.10651 | 0.197387 |
|  |  | Weighted median | |  | 25.22212 | 0.237672 |
|  |  | Inverse variance weighted | |  |  |  |
| genus Ruminococcaceae UCG003 | Betaine | MR Egger | 0.002733 | 0.73649 | 3.575269 | 0.466526 |
|  |  | Weighted median | |  | 3.705452 | 0.592553 |
|  |  | Inverse variance weighted | |  |  |  |
| genus Ruminococcaceae UCG003 | Bradykinin | MR Egger | 0.010866 | 0.773305 | 3.112008 | 0.539259 |
|  |  | Weighted median | |  | 3.207002 | 0.668107 |
|  |  | Inverse variance weighted | |  |  |  |
| genus Desulfovibrio | Ratio of bisallylic groups to total fatty acids | MR Egger | -0.01607 | 0.368129 | 6.276996 | 0.616235 |
|  |  | Weighted median | |  | 7.186661 | 0.617692 |
|  |  | Inverse variance weighted | |  |  |  |
|  | Cholesterol in medium HDL | MR Egger | 0.001738 | 0.730177 | 8.095112 | 0.52459 |
|  |  | Weighted median | |  | 8.221715 | 0.60719 |
|  |  | Inverse variance weighted | |  |  |  |
| genus Eubacterium eligens group | Myo-inositol | MR Egger | 0.008257 | 0.361257 | 0.504355 | 0.477593 |
|  |  | Weighted median | |  | 2.965772 | 0.226982 |
|  |  | Inverse variance weighted | |  |  |  |
|  | Alanine | MR Egger | 0.008809 | 0.345796 | 0.750718 | 0.386249 |
|  |  | Weighted median | |  | 3.494061 | 0.174291 |
|  |  | Inverse variance weighted | |  |  |  |
|  | Indolepropionate | MR Egger | 0.001722 | 0.900941 | 0.690466 | 0.406006 |
|  |  | Weighted median | |  | 0.715074 | 0.699397 |
|  |  | Inverse variance weighted | |  |  |  |
| genus Ruminococcaceae UCG014 | Alanine | MR Egger | 0.003646 | 0.407451 | 0.890408 | 0.925933 |
|  |  | Weighted median | |  | 1.745505 | 0.883118 |
|  |  | Inverse variance weighted | |  |  |  |
|  | Cholesterol in medium HDL | MR Egger | 0.002068 | 0.692299 | 12.78235 | 0.172705 |
|  |  | Weighted median | |  | 13.0196 | 0.222578 |
|  |  | Inverse variance weighted | |  |  |  |
| Glycerate | Anti-Influenza virus subtype H1N1 IgG levels | MR Egger | -0.09956 | 0.326954 | 2.809504 | 0.590193 |
|  |  | Weighted median | |  | 4.054866 | 0.541544 |
|  |  | Inverse variance weighted | |  |  |  |
| Heptanoate |  | MR Egger | 0.064687 | 0.197668 | 4.824479 | 0.939368 |
|  |  | Weighted median | |  | 6.704392 | 0.876514 |
|  |  | Inverse variance weighted | |  |  |  |
| Betaine |  | MR Egger | -0.07409 | 0.511093 | 6.393454 | 0.171629 |
|  |  | Weighted median | |  | 7.223122 | 0.204568 |
|  |  | Inverse variance weighted | |  |  |  |
| DSGEGDFXAEGGGVR* |  | MR Egger | 0.207276 | 0.243726 | 2.961005 | 0.564372 |
|  |  | Weighted median | |  | 4.826714 | 0.437392 |
|  |  | Inverse variance weighted | |  |  |  |
| ADSGEGDFXAEGGGVR* |  | MR Egger | -0.00057 | 0.995431 | 4.881673 | 0.18067 |
|  |  | Weighted median | |  | 4.881736 | 0.299649 |
|  |  | Inverse variance weighted | |  |  |  |
| 1-arachidonoylglycerophosphoinositol* |  | MR Egger | -0.04906 | 0.526501 | 5.105898 | 0.824981 |
|  |  | Weighted median | |  | 5.539952 | 0.852322 |
|  |  | Inverse variance weighted | |  |  |  |
| Bradykinin |  | MR Egger | 0.055908 | 0.368555 | 3.003552 | 0.808401 |
|  |  | Weighted median | |  | 3.948481 | 0.785692 |
|  |  | Inverse variance weighted | |  |  |  |
| 2-palmitoylglycerophosphocholine* |  | MR Egger | -0.04516 | 0.492239 | 2.759266 | 0.97308 |
|  |  | Weighted median | |  | 3.271704 | 0.974288 |
|  |  | Inverse variance weighted | |  |  |  |
| Alanine |  | MR Egger | 0.028949 | 0.687863 | 13.99911 | 0.449777 |
|  |  | Weighted median | |  | 14.16738 | 0.512874 |
|  |  | Inverse variance weighted | |  |  |  |
| Apolipoprotein A-I |  | MR Egger | -0.00393 | 0.905119 | 20.93827 | 0.789171 |
|  |  | Weighted median | |  | 20.95275 | 0.827313 |
|  |  | Inverse variance weighted | |  |  |  |
| Free cholesterol |  | MR Egger | 0.054593 | 0.084152 | 45.72783 | 0.153759 |
|  |  | Weighted median | |  | 49.62095 | 0.098184 |
|  |  | Inverse variance weighted | |  |  |  |
| Total cholesterol in HDL |  | MR Egger | 0.003762 | 0.89677 | 28.89988 | 0.522861 |
|  |  | Weighted median | |  | 28.917 | 0.573556 |
|  |  | Inverse variance weighted | |  |  |  |
| Free cholesterol to total lipids ratio in large LDL |  | MR Egger | -0.00582 | 0.538346 | 219.7271 | 0.06867 |
|  |  | Weighted median | |  | 220.1665 | 0.072675 |
|  |  | Inverse variance weighted | |  |  |  |
| Concentration of VLDL particles |  | MR Egger | 0.005449 | 0.657288 | 137.0474 | 0.506904 |
|  |  | Weighted median | |  | 137.2451 | 0.526175 |
|  |  | Inverse variance weighted | |  |  |  |
| Triglycerides in very large HDL |  | MR Egger | 0.001813 | 0.881905 | 130.3908 | 0.243517 |
|  |  | Weighted median | |  | 130.4149 | 0.263435 |
|  |  | Inverse variance weighted | |  |  |  |
| Guanosine | Anti-Influenza virus subtype H3N2 IgG levels | MR Egger | 0.011 | 0.928438 | 4.825127 | 0.437593 |
|  |  | Weighted median | |  | 4.834043 | 0.565268 |
|  |  | Inverse variance weighted | |  |  |  |
| Myo-inositol |  | MR Egger | -0.03316 | 0.258032 | 12.55779 | 0.944766 |
|  |  | Weighted median | |  | 13.90602 | 0.929531 |
|  |  | Inverse variance weighted | |  |  |  |
| Indoleacetate |  | MR Egger | 0.046347 | 0.543413 | 3.566374 | 0.613369 |
|  |  | Weighted median | |  | 3.990968 | 0.677899 |
|  |  | Inverse variance weighted | |  |  |  |
| Alanine |  | MR Egger | 0.051186 | 0.45925 | 14.74638 | 0.194405 |
|  |  | Weighted median | |  | 15.53495 | 0.213476 |
|  |  | Inverse variance weighted | |  |  |  |
| Indolepropionate |  | MR Egger | 0.012654 | 0.851179 | 3.720025 | 0.714503 |
|  |  | Weighted median | |  | 3.758389 | 0.807148 |
|  |  | Inverse variance weighted | |  |  |  |
| phenylalanylleucine |  | MR Egger | -0.31314 | 0.393496 | 6.59E-06 | 0.997952 |
|  |  | Weighted median | |  | 1.977905 | 0.371966 |
|  |  | Inverse variance weighted | |  |  |  |
| MDC:%CD1c+ |  | MR Egger | 0.068771 | 0.802547 | 1.265398 | 0.260632 |
|  |  | Weighted median | |  | 1.395382 | 0.497733 |
|  |  | Inverse variance weighted | |  |  |  |
| Acetoacetate |  | MR Egger | 0.034422 | 0.700896 | 12.74141 | 0.238491 |
|  |  | Weighted median | |  | 12.94055 | 0.297229 |
|  |  | Inverse variance weighted | |  |  |  |
| Ratio of bisallylic groups to total fatty acids |  | MR Egger | 0.008547 | 0.791693 | 20.90012 | 0.342346 |
|  |  | Weighted median | |  | 20.97904 | 0.398367 |
|  |  | Inverse variance weighted | |  |  |  |
| Citrate |  | MR Egger | -0.06317 | 0.462275 | 4.016001 | 0.969447 |
|  |  | Weighted median | |  | 4.596142 | 0.970128 |
|  |  | Inverse variance weighted | |  |  |  |
| Lactate |  | MR Egger | -0.07777 | 0.251692 | 8.074382 | 0.326081 |
|  |  | Weighted median | |  | 9.87482 | 0.273922 |
|  |  | Inverse variance weighted | |  |  |  |
| Acetone |  | MR Egger | 0.018549 | 0.585468 | 23.70246 | 0.822495 |
|  |  | Weighted median | |  | 24.00625 | 0.844189 |
|  |  | Inverse variance weighted | |  |  |  |
| Cholesterol in medium HDL |  | MR Egger | -0.01489 | 0.224254 | 158.6552 | 0.190823 |
|  |  | Weighted median | |  | 160.2965 | 0.182125 |
|  |  | Inverse variance weighted | |  |  |  |
| Triglycerides in medium LDL |  | MR Egger | 0.013264 | 0.248896 | 198.9475 | 0.077978 |
|  |  | Weighted median | |  | 200.4958 | 0.074678 |
|  |  | Inverse variance weighted | |  |  |  |

| **Supplementary Table 5. Mendelian randomization models estimate the causal effects of blood metabolites on anti-influenza H1N1 IgG level** | | | | | | | | |
| --- | --- | --- | --- | --- | --- | --- | --- | --- |
| Exposure | Outcome | Method | Nsnp | BETA | SE | Pval | OR (95% CI) |  |
| Glycerate | Anti-Influenza virus subtype H1N1 IgG levels | MR Egger | 6 | 11.13402 | 5.224517 | 0.100083 | 68461.1254 (2.4448, 1917091116.1457) | |
|  |  | Weighted median | 6 | 5.866695 | 2.792363 | 0.035643 | 353.0800 (1.4824, 84097.0198) | |
|  |  | Inverse variance weighted | 6 | 5.767226 | 2.041512 | 0.004728 | 319.6499 (5.8466, 17476.1052) | |
| Heptanoate |  | MR Egger | 13 | -8.19036 | 4.006547 | 0.065623 | 0.0003 (0.0000, 0.7135) | |
|  |  | Weighted median | 13 | -4.417581 | 2.227237 | 0.047319 | 0.0121 (0.0002, 0.9491) | |
|  |  | Inverse variance weighted | 13 | -3.161188 | 1.611923 | 0.049864 | 0.0424 (0.0018, 0.9982) | |
| Betaine |  | MR Egger | 6 | -0.577313 | 6.014073 | 0.928143 | 0.5614 (0.0000, 73885.4141) | |
|  |  | Weighted median | 6 | -1.291203 | 2.44065 | 0.596777 | 0.2749 (0.0023, 32.8671) | |
|  |  | Inverse variance weighted | 6 | -4.618855 | 2.061343 | 0.025045 | 0.0099 (0.0002, 0.5607) | |
| DSGEGDFXAEGGGVR* |  | MR Egger | 6 | -7.290257 | 4.038954 | 0.145392 | 0.0007 (0.0000, 1.8703) | |
|  |  | Weighted median | 6 | -0.882557 | 1.085141 | 0.41604 | 0.4137 (0.0493, 3.4706) | |
|  |  | Inverse variance weighted | 6 | -1.881847 | 0.796855 | 0.018197 | 0.1523 (0.0319, 0.7261) | |
| ADSGEGDFXAEGGGVR* |  | MR Egger | 5 | -1.910753 | 2.007363 | 0.411385 | 0.1480 (0.0029, 7.5661) | |
|  |  | Weighted median | 5 | -2.132614 | 0.884653 | 0.015923 | 0.1185 (0.0209, 0.6712) | |
|  |  | Inverse variance weighted | 5 | -1.921678 | 0.83958 | 0.022088 | 0.1464 (0.0282, 0.7587) | |
| 1-arachidonoylglycerophosphoinositol* |  | MR Egger | 11 | 0.122685 | 4.251313 | 0.977607 | 1.1305 (0.0003, 4699.7206) | |
|  |  | Weighted median | 11 | -1.833272 | 1.63323 | 0.261658 | 0.1599 (0.0065, 3.9269) | |
|  |  | Inverse variance weighted | 11 | -2.562125 | 1.211185 | 0.034397 | 0.0771 (0.0072, 0.8284) | |
| Bradykinin |  | MR Egger | 8 | -1.321793 | 0.736796 | 0.122974 | 0.2667 (0.0629, 1.1301) | |
|  |  | Weighted median | 8 | -0.860788 | 0.398153 | 0.030622 | 0.4228 (0.1938, 0.9227) | |
|  |  | Inverse variance weighted | 8 | -0.679308 | 0.325612 | 0.036956 | 0.5070 (0.2678, 0.9597) | |
| 2-palmitoylglycerophosphocholine* |  | MR Egger | 11 | -1.523398 | 4.197583 | 0.72504 | 0.2180 (0.0000, 815.5508) | |
|  |  | Weighted median | 11 | -4.193938 | 1.821585 | 0.021315 | 0.0151 (0.0004, 0.5360) | |
|  |  | Inverse variance weighted | 11 | -4.331593 | 1.493522 | 0.003729 | 0.0131 (0.0007, 0.2455) | |
| Alanine |  | MR Egger | 16 | 0.303209 | 0.891798 | 0.738906 | 1.3542 (0.2358, 7.7767) | |
|  |  | Weighted median | 16 | 0.351285 | 0.428634 | 0.412474 | 1.4209 (0.6133, 3.2917) | |
|  |  | Inverse variance weighted | 16 | 0.648469 | 0.294771 | 0.027814 | 1.9126 (1.0733, 3.4083) | |
| Apolipoprotein A-I |  | MR Egger | 29 | 0.397006 | 0.298606 | 0.194798 | 1.4874 (0.8284, 2.6705) | |
|  |  | Weighted median | 29 | 0.355028 | 0.210457 | 0.091615 | 1.4262 (0.9442, 2.1544) | |
|  |  | Inverse variance weighted | 29 | 0.365789 | 0.147833 | 0.013348 | 1.4417 (1.0790, 1.9262) | |
| Free cholesterol |  | MR Egger | 39 | -0.147151 | 0.270733 | 0.590027 | 0.8632 (0.5077, 1.4674) | |
|  |  | Weighted median | 39 | 0.195569 | 0.18958 | 0.302265 | 1.2160 (0.8386, 1.7632) | |
|  |  | Inverse variance weighted | 39 | 0.270382 | 0.137729 | 0.049628 | 1.3105 (1.0004, 1.7166) | |
| Total cholesterol in HDL |  | MR Egger | 32 | 0.322678 | 0.264851 | 0.232591 | 1.3808 (0.8217, 2.3205) | |
|  |  | Weighted median | 32 | 0.286875 | 0.220034 | 0.19231 | 1.3323 (0.8655, 2.0506) | |
|  |  | Inverse variance weighted | 32 | 0.352068 | 0.140337 | 0.012116 | 1.4220 (1.0801, 1.8722) | |
| Free cholesterol to total lipids ratio in large LDL |  | MR Egger | 192 | -0.19028 | 0.207624 | 0.360587 | 0.8267 (0.5503, 1.2419) | |
|  |  | Weighted median | 192 | -0.464391 | 0.244478 | 0.057496 | 0.6285 (0.3892, 1.0149) | |
|  |  | Inverse variance weighted | 192 | -0.284912 | 0.139561 | 0.041202 | 0.7521 (0.5721, 0.9887) | |
| Concentration of VLDL particles |  | MR Egger | 140 | 0.209002 | 0.272757 | 0.444832 | 1.2324 (0.7221, 2.1035) | |
|  |  | Weighted median | 140 | 0.341209 | 0.265046 | 0.19797 | 1.4066 (0.8367, 2.3648) | |
|  |  | Inverse variance weighted | 140 | 0.30812 | 0.157166 | 0.04994 | 1.3609 (1.0001, 1.8518) | |
| Triglycerides in very large HDL |  | MR Egger | 122 | 0.494434 | 0.232371 | 0.035402 | 1.6396 (1.0398, 2.5854) | |
|  |  | Weighted median | 122 | 0.453212 | 0.238482 | 0.057381 | 1.5734 (0.9859, 2.5109) | |
|  |  | Inverse variance weighted | 122 | 0.520641 | 0.151067 | 0.000568 | 1.6831 (1.2518, 2.2631) | |

| **Supplementary Table 6. Mendelian randomization models estimate the causal effects of blood metabolites on anti-influenza H3N2 IgG level** | | | | | | | |
| --- | --- | --- | --- | --- | --- | --- | --- |
| Exposure | Outcome | Method | Nsnp | BETA | SE | Pval | OR (95% CI) |
| Guanosine | Anti-Influenza virus subtype H3N2 IgG levels | MR Egger | 7 | -2.8031 | 2.11966 | 0.243284 | 0.061 (0.00, 3.863) |
|  |  | Weighted median | 7 | -2.66469 | 0.927544 | 0.004068 | 0.070 (0.01, 0.429) |
|  |  | Inverse variance weighted | 7 | -2.61327 | 0.672005 | 0.000101 | 0.073 (0.02, 0.274) |
| Myo-inositol |  | MR Egger | 24 | -0.05485 | 2.085776 | 0.979256 | 0.947 (0.01, 56.445) |
|  |  | Weighted median | 24 | -1.37114 | 1.561751 | 0.379971 | 0.254 (0.01, 5.419) |
|  |  | Inverse variance weighted | 24 | -2.12653 | 1.080344 | 0.049024 | 0.119 (0.01, 0.991) |
| Indoleacetate |  | MR Egger | 7 | 1.814712 | 2.172405 | 0.441598 | 6.139 (0.08, 433.818) |
|  |  | Weighted median | 7 | 2.765612 | 1.588513 | 0.081682 | 15.889 (0.70, 357.487) |
|  |  | Inverse variance weighted | 7 | 2.979788 | 1.233856 | 0.015734 | 19.684 (1.75, 220.996) |
| Alanine |  | MR Egger | 13 | -8.85003 | 6.14263 | 0.177506 | 0.000 (0.00, 24.277) |
|  |  | Weighted median | 13 | -4.17364 | 2.732045 | 0.126596 | 0.015 (0.00, 3.258) |
|  |  | Inverse variance weighted | 13 | -4.44278 | 2.132898 | 0.037253 | 0.012 (0.00, 0.769) |
| Indolepropionate |  | MR Egger | 8 | 1.748244 | 2.430994 | 0.499088 | 5.745 (0.04, 673.840) |
|  |  | Weighted median | 8 | 2.062684 | 1.233949 | 0.094601 | 7.867 (0.70, 88.343) |
|  |  | Inverse variance weighted | 8 | 2.189392 | 0.914871 | 0.016706 | 8.930 (1.48, 53.653) |
| phenylalanylleucine |  | MR Egger | 3 | 5.139078 | 5.146685 | 0.500471 | 170.558 (0.00, 4100339.475) |
|  |  | Weighted median | 3 | -1.37822 | 1.114955 | 0.216414 | 0.252 (0.02, 2.241) |
|  |  | Inverse variance weighted | 3 | -1.9854 | 0.908683 | 0.028895 | 0.137 (0.02, 0.815) |
| MDC:%CD1c+ |  | MR Egger | 3 | 0.181959 | 1.912447 | 0.939611 | 1.200 (0.02, 50.925) |
|  |  | Weighted median | 3 | 0.682393 | 0.461477 | 0.139216 | 1.979 (0.80, 4.889) |
|  |  | Inverse variance weighted | 3 | 0.781267 | 0.356596 | 0.028459 | 2.184 (1.08, 4.394) |
| Acetoacetate |  | MR Egger | 12 | -1.4312 | 0.857419 | 0.126037 | 0.239 (0.04, 1.283) |
|  |  | Weighted median | 12 | -0.73754 | 0.474762 | 0.120306 | 0.478 (0.18, 1.213) |
|  |  | Inverse variance weighted | 12 | -1.12628 | 0.35987 | 0.00175 | 0.324 (0.16, 0.656) |
| Ratio of bisallylic groups to total fatty acids |  | MR Egger | 21 | -0.38357 | 0.2142 | 0.089291 | 0.681 (0.44, 1.037) |
|  |  | Weighted median | 21 | -0.29649 | 0.178546 | 0.096798 | 0.743 (0.52, 1.055) |
|  |  | Inverse variance weighted | 21 | -0.33896 | 0.131584 | 0.009994 | 0.713 (0.55, 0.922) |
| Citrate |  | MR Egger | 13 | 0.166524 | 1.023816 | 0.873743 | 1.181 (0.15, 8.786) |
|  |  | Weighted median | 13 | -0.70543 | 0.361088 | 0.050745 | 0.494 (0.24, 1.002) |
|  |  | Inverse variance weighted | 13 | -0.58212 | 0.286552 | 0.042208 | 0.559 (0.31, 0.980) |
| Lactate |  | MR Egger | 9 | -0.28766 | 0.741892 | 0.709731 | 0.750 (0.17, 3.211) |
|  |  | Weighted median | 9 | -1.50875 | 0.563107 | 0.007377 | 0.221 (0.07, 0.667) |
|  |  | Inverse variance weighted | 9 | -1.05782 | 0.427007 | 0.013238 | 0.347 (0.15, 0.802) |
| Acetone |  | MR Egger | 33 | -1.61755 | 0.877414 | 0.074829 | 0.198 (0.03, 1.108) |
|  |  | Weighted median | 33 | -0.63933 | 0.637477 | 0.315909 | 0.528 (0.15, 1.841) |
|  |  | Inverse variance weighted | 33 | -1.20343 | 0.453148 | 0.007914 | 0.300 (0.12, 0.730) |
| Cholesterol in medium HDL |  | MR Egger | 146 | -0.06015 | 0.287671 | 0.834666 | 0.942 (0.53, 1.655) |
|  |  | Weighted median | 146 | -0.16932 | 0.277358 | 0.541536 | 0.844 (0.49, 1.454) |
|  |  | Inverse variance weighted | 146 | -0.34178 | 0.17209 | 0.04703 | 0.711 (0.50, 0.996) |
| Triglycerides in medium LDL |  | MR Egger | 174 | 0.085636 | 0.256246 | 0.738639 | 1.089 (0.65, 1.800) |
|  |  | Weighted median | 174 | -0.03312 | 0.247954 | 0.893735 | 0.967 (0.59, 1.573) |
|  |  | Inverse variance weighted | 174 | 0.317272 | 0.160085 | 0.047491 | 1.373 (1.00, 1.880) |

| **Supplementary Table 7. Mendelian randomization models estimate the causal effects of gut microbiota on blood metabolites** | | | | | | |  |  |
| --- | --- | --- | --- | --- | --- | --- | --- | --- |
| Exposure | Outcome | Method | Nsnp | BETA | SE | Pval | OR (95% CI) |  |
| family Erysipelotrichaceae | Bradykinin | MR Egger | 4 | -0.42578708 | 0.714744562 | 0.6117986 | 0.653 (0.161, 2.651) | |
|  |  | Weighted median | 4 | 0.22873098 | 0.148600686 | 0.1237476 | 1.257 (0.936, 1.689) | |
|  |  | Inverse variance weighted | 4 | 0.35303897 | 0.129041712 | 0.00622191 | 1.423 (1.105, 1.833) | |
| family Erysipelotrichaceae | Triglycerides in very large HDL | MR Egger | 13 | -0.07501836 | 0.101223958 | 0.47414975 | 0.928 (0.761, 1.131) | |
|  |  | Weighted median | 13 | 0.03750436 | 0.03121829 | 0.22961216 | 1.038 (0.974, 1.106) | |
|  |  | Inverse variance weighted | 13 | 0.04853456 | 0.024329847 | 0.04605852 | 1.050 (1.001, 1.101) | |
| genus Barnesiella | Heptanoate | MR Egger | 4 | -0.0229008 | 0.10036359 | 0.84071351 | 0.977 (0.803, 1.190) | |
|  |  | Weighted median | 4 | 0.05487732 | 0.029768174 | 0.06525754 | 1.056 (0.997, 1.120) | |
|  |  | Inverse variance weighted | 4 | 0.0465935 | 0.023310775 | 0.04563036 | 1.048 (1.001, 1.097) | |
| genus Barnesiella | ADSGEGDFXAEGGGVR* | MR Egger | 4 | -0.23041936 | 0.130181793 | 0.21874989 | 0.794 (0.615, 1.025) | |
|  |  | Weighted median | 4 | -0.07736779 | 0.054327628 | 0.15441849 | 0.926 (0.832, 1.030) | |
|  |  | Inverse variance weighted | 4 | -0.09668348 | 0.043761422 | 0.02715161 | 0.908 (0.833, 0.989) | |
| genus Ruminococcaceae UCG002 | Concentration of VLDL particles | MR Egger | 22 | 0.05063963 | 0.047755916 | 0.30160816 | 1.052 (0.958, 1.155) | |
|  |  | Weighted median | 22 | 0.03734406 | 0.024370859 | 0.12544237 | 1.038 (0.990, 1.089) | |
|  |  | Inverse variance weighted | 22 | 0.03719223 | 0.017417438 | 0.0327329 | 1.038 (1.003, 1.074) | |
| genus Ruminococcaceae UCG003 | Betaine | MR Egger | 6 | -0.00208488 | 0.106292277 | 0.98529022 | 0.998 (0.810, 1.229) | |
|  |  | Weighted median | 6 | 0.03725142 | 0.020519007 | 0.06945341 | 1.038 (0.997, 1.081) | |
|  |  | Inverse variance weighted | 6 | 0.0358144 | 0.016269259 | 0.02771095 | 1.036 (1.004, 1.070) | |
| genus Ruminococcaceae UCG003 | Bradykinin | MR Egger | 6 | -0.34237097 | 0.495594213 | 0.52767086 | 0.710 (0.269, 1.876) | |
|  |  | Weighted median | 6 | -0.11103991 | 0.095594386 | 0.24540874 | 0.895 (0.742, 1.079) | |
|  |  | Inverse variance weighted | 6 | -0.19129698 | 0.073169869 | 0.00893783 | 0.826 (0.716, 0.953) | |
| genus Desulfovibrio | Ratio of bisallylic groups to total fatty acids | MR Egger | 10 | 0.28366301 | 0.171002344 | 0.13573538 | 1.328 (0.95, 1.857) | |
|  |  | Weighted median | 10 | 0.13463992 | 0.077843464 | 0.08369748 | 1.144 (0.98, 1.333) | |
|  |  | Inverse variance weighted | 10 | 0.13058197 | 0.058998984 | 0.02687752 | 1.139 (1.01, 1.279) | |
|  | Cholesterol in medium HDL | MR Egger | 11 | 0.03493326 | 0.050121737 | 0.503426 | 1.036 (0.93, 1.142) | |
|  |  | Weighted median | 11 | 0.04266799 | 0.021560935 | 0.04782177 | 1.044 (1.00, 1.089) | |
|  |  | Inverse variance weighted | 11 | 0.05176372 | 0.016576395 | 0.00179178 | 1.053 (1.01, 1.088) | |
| genus Eubacterium eligens group | Myo-inositol | MR Egger | 3 | -0.04789046 | 0.073402256 | 0.63197802 | 0.953 (0.82, 1.101) | |
|  |  | Weighted median | 3 | 0.05021249 | 0.028879779 | 0.08209229 | 1.051 (0.99, 1.113) | |
|  |  | Inverse variance weighted | 3 | 0.06308205 | 0.023884968 | 0.00826428 | 1.065 (1.01, 1.116) | |
|  | Alanine | MR Egger | 3 | -0.06353023 | 0.074565406 | 0.5507642 | 0.938 (0.81, 1.086) | |
|  |  | Weighted median | 3 | 0.04431271 | 0.02862289 | 0.12158464 | 1.045 (0.98, 1.106) | |
|  |  | Inverse variance weighted | 3 | 0.05553296 | 0.026188215 | 0.03396116 | 1.057 (1.00, 1.113) | |
|  | Indolepropionate | MR Egger | 3 | 0.06156316 | 0.148805247 | 0.75027022 | 1.063 (0.79, 1.424) | |
|  |  | Weighted median | 3 | 0.10036972 | 0.05211837 | 0.05412895 | 1.106 (0.99, 1.224) | |
|  |  | Inverse variance weighted | 3 | 0.084049 | 0.039954093 | 0.03540993 | 1.088 (1.00, 1.176) | |
| genus Ruminococcaceae UCG014 | Alanine | MR Egger | 6 | -0.10048126 | 0.060250802 | 0.17069818 | 0.904 (0.80, 1.018) | |
|  |  | Weighted median | 6 | -0.03652971 | 0.020356135 | 0.07272851 | 0.964 (0.92, 1.003) | |
|  |  | Inverse variance weighted | 6 | -0.04664336 | 0.015506933 | 0.00263057 | 0.954 (0.92, 0.984) | |
|  | Cholesterol in medium HDL | MR Egger | 11 | 0.02229836 | 0.063906971 | 0.73517404 | 1.023 (0.90, 1.159) | |
|  |  | Weighted median | 11 | 0.03696331 | 0.027761146 | 0.18303235 | 1.038 (0.98, 1.096) | |
|  |  | Inverse variance weighted | 11 | 0.04662395 | 0.022288014 | 0.03644887 | 1.048 (1.00, 1.095) | |
